# Abuses of the Stomach

**Published:** 1878-04

**Authors:** Ben. H. Pratt


// THE BISTOURY.

Vol. XIV. ELMIRA, N. Y., APRIL, 1878. No. 1.

### ABUSES OF THE STOMACH;

### *Or, THE NEED OF CIBARIOUS REFORM.*

BEN. H. PRATT.

The average man spends one hour of the twenty-four at his meals, and for the sake of enjoying that one hour, ninety-nine men out of every hundred make the other twenty-three hours a season of misery. It is a singular fact, but none the less a fact, that men, for the sake of having their palates tickled twenty minutes, three times a day, will endure untold misery between those seasons of eating and drinking. It has come to pass that people won't eat plain food any longer, if they can help it; and they eat, not to satisfy hunger—not to nourish their bodies—not to strengthen their systems—not to fit them for their several duties—but to titillate their organs of taste, regardless of consequences, whether immediate bodily discomforts or the entailment of chronic diseases from which they must suffer through a long life-time.

How many people are there who are not hourly reminded that they have a stomach, because of the struggles of that organ to resist the incursions made upon it by enemies that never ought to have been introduced to it? A man will never know that he has a stomach—except upon hearsay evidence—unless he abuses that stomach. Not to be aware of the presence of any organ in the system, is pretty conclusive evidence that no organic disease exists there. When one complains of having this or that peculiar sensation somewhere or anywhere, it's good evidence that something is wrong there. Yet men will go on from year to year, with a growing trouble here and there, constantly reminding them of an organ out of order, and yet take no sensible means to be rid of the trouble. We claim that a normal man—a healthy man—might live his allotted seventy years and never, except through evidence other than his own sensations, know that he had a brain, or a set of nerves, or a pair of lungs, or a liver, or kidneys, or a stomach, or bowels, or any of the multitude of organs which constitute his internality. Children have little or no knowledge of their own as to the complexity of their internal organism. We all remember well our first impressions of our inner selves. When we began to think about these things at all, we regarded our head as a big hollow ball of bone, with skin stretched over it, just as a cover is stretched over a base ball. We imagined our trunk to be a big sack, with a couple of flexible pipes leading into it, and a couple more leading out of it. When we ate a big dinner we thought we just filled up the big cavity, and as the filling kept working off we had to keep up the supply. We felt hunger, and explained it upon the ground that the big sack was getting empty, and needed filling up. We once heard an old man tell how hungry he was one time, having gone a whole day without eating. "Why," said he, "I was that empty that when I began to eat I could feel and actually hear every mouthful I swallowed fall down kerslap on the bottom of my belly." Children often say, when they have eaten a big dinner, that "they are full clear up to their necks." Now this man and these children were healthy, otherwise they would have learned, long before, that they had stomachs to ache, and bowels to gripe, and that other organs were filling up those great spaces which we call the thoracic and abdominal cavities. In fact, the more trouble we have inside, the more we begin to learn about our insides, and the growing intelligence of the people upon their interior make-up, is due more to their aches and pains, their stitches and catches, their sorenesses and distresses, than to their zeal for anatomical knowledge or their physiological research. Take a man who has a kidney difficulty, for instance: why, he can rattle off technical terms to his doctor so glibly as to impress the unfortunate by-stander, who don't know whether he has any kidneys or not—and rather thinks he hasn't—that he's a wonderful student of anatomy. So with a nervous person—so with a consumptive—so with a dyspeptic—so with a bilious fellow—so with a paralytic—so with one of these head ache chaps, who are eternally talking about their cephalic appendage--so with the neuralgic—so with the rheumatic—so with the heart diseased—any one of them, with big words, will stump a healthy doctor, who would n't know he had nerves and lungs and stomach and liver and brains and kidneys and bowels and a heart and all these things, if he had n't read about them in the way of business—and believes it then only as a sort of tradition of some of those old Esculapians, who dug into men, and showed up these organs. Job, we wager, knew more about eruptive diseases than any of the doctors of his time, and no one, except He who cured him, knew anything about the leprosy, compared with Naaman the Syrian. Now all this goes to show that people who are ailing, or have spells, or suffer from the thousand and one different troubles that the human system is heir to, know a good deal about themselves, and how they ought to treat themselves, and yet are continually doing the very things, day after day, they ought not to do—actually abusing themselves—making themselves miserable—keeping themselves in a continual state of discomfort and almost unendurability until they get beyond a curing or helping point, and then down they go into life-long invalidism.

But we did n't purpose going over so much ground. The abuse of the stomach was what we purposed pointing out, and to show how much sympathy is wasted on dyspeptics, and those who can't talk five minutes without telling you in what a fearful condition their stomachs are, and how they suffer from that organ's persecution of them. We can't seriously mourn with one of these, especially if we sit at his table and see him eat—or know the habits of his family, and see his itemized grocer's and butcher's and confectioner's bills. It's enough to make a man hate a dyspeptic, even though he may be his best friend, to go into an eating-house with him, and see him take "a little bite of something just to stay his stomach before he goes to bed." Fried oysters, with condiment enough thrust into their composition, while being cooked, to make a healthy stomach peel its coat, are further treated, upon his plate, to butter and mustard and enough of those other mucous membrane destroyers, to eat their way through the sole of a cowhide boot. These are hurriedly hustled into the delicate stomach and carried to bed to do their gastrophagic work. It seems as though the dyspeptic is possessed of a devil that is continually tempting him to hurry up and get through with life, that he may join his impship in the realm Plutonic. The mania for high living is becoming one of the most terrible life scourges the world has ever known, second only to intemperance. Indeed it is a question whether more direct harm to the race is not being accomplished by the intemperance of eating than by the intemperance of drinking. Looking at the matter fairly and squarely, the former seems the less deserving of sympathy, from the fact that excesses in eating are not surrounded by half the fascinations that tempt men to excess in drink. The former indulgence is even followed by dullness, stupidity, and more or less of suffering while the latter is succeeded by unnatural brilliancy, temporary ecstacy, and an oblivion of all those ills which tend to make men miserable. Indeed we have larger sympathy for the drunkard than for the glutton and the epicurean, yet the latter will rise, in sleek skinned beamfulness and oleaginous raptures, from his stupefying banquet, and with conventional platitudes and superficialities, denounce intemperance, while with one hand upon his oppressed abdomen, and the other supporting his over-fed corporosity, will, between his oft-belchings, detail the sinfulness and the horrors of wine bibbing. Drunkenness asks no odds of gluttony, so far as the wrongfulness of the excess is concerned. The difference is in the results. The drunken surely harms himself, the other may harm himself, his family and his neighbor. It ill becomes him who yields to an appetite for food that he knows is undermining his constitution, to berate him who yields to an appetite for drink that is no more and no less a destroyer of his physical power.

If men would live plainly, they would live longer, be healthier, enjoy life better, and rarely be reminded of the possession of organs because of the derangement of those organs. Too much food, too rich food, food too often partaken of, food stuffed in for the sake of gratifying the palate rather than satisfying the hunger—these give rise to more and greater evils than most men will grant, simply because most men are guilty of the excesses we mention. As the ancient epicureans, for the sake of gratifying their appetites *ad libitum*, regarded the spew-bowl a necessary article of dinner table furniture, into which they could disgorge what they had eaten, and then fill up again—who lived to eat, and never believed themselves happy except while indulging their appetites—so now-a-days we, though not quite so enslaved by our palates as to resort to the spew-bowl, do nearly the same thing in a less disgusting manner—have our dinner-pills ready to take before and after a season of stuffing, in order to artificially digest what we have wasted upon our stomachs. The ancient did the more sensible thing, however, preferring to throw away what he could n't use, while we devise methods for forcing our systems into clearing off the rubbish which nature refuses to scavenge. Medical science and art are called into requisition to provide means for enabling us to eat all we can, and of as rich food as we may, without killing ourselves outright. Sauces prepared with occult skill, are provided for the purpose of antidoting the bad effects of certain sorts of food which men insist upon eating because a depraved appetite demands it. The gastic juices of the human and other species are analyzed in order that men may make an artificial solvent of such power as to artificially digest the most indigestible food. The pepsin bottle upon the table of the man who has not the will power to resist the temptations of the feast, is a terrible commentary upon the weakness of our race.

To deliberately do that which we know is injuring health, begetting disease, heaping up misery for the future, and taking years from our allotted life-time, is admitted by every one, not only a supreme folly, but a crime against nature and against God; and yet hundreds, aye thousands, are daily doing this, and yet admitting their guilt. Self-denial is a bug bear. We think we can't deny ourselves because we have never honestly tried. One is astonished to find how easy it is to do many things which he once deemed impossibilities. Compelled to denial, we accomplish it without more than a passing struggle. To make up one's mind to do a thing is more difficult than to do the thing we 're determined to do. To confess that we are unable to resist a temptation implies that we never really intended to resist it.

The world needs a temperance revival for eaters as well as for drinkers. We need societies to encourage the substitution of plain, well-cooked food, for that which has been introduced into our *cuisine.* Plain living needs to be popularized—made fashionable, if we may so say. Let us eat to satisfy hunger, and stop eating when hunger is satisfied. Let us go without eating until plain food tastes good, and we will be surprised to find how much better plain food, sauced with hunger is, than the richest viands, partaken of for the sake of tickling the sense of taste. Our cooks could do much for us in the way of a reformation, if they only had the courage to stand out for it. Our wives and our mothers and our female relatives, under the impression that they are doing us a kindness in treating us to appetizing viands, are, in reality, planting within us the seeds of disease and infirmity and early death. It is a mistaken love that treats the child to dainties and the man to those exquisite emanations of the *cuisine* which tempt him to over-indulgence. The day that marks a change in our cooking will mark a change in our health and happiness, and it only needs the adoption of plain living by the leaders of society to redeem the race from fast-approaching invalidity.

